# Inequities in implementation: an EDI analysis of compliance with the European working time directive

**DOI:** 10.1192/j.eurpsy.2026.10355

**Published:** 2026-07-31

**Authors:** L. Tarchi

**Affiliations:** Department of Health Sciences, University of Florence, Florence, Italy

## Abstract

**Abstract:**

The European Working Time Directive (EWTD) mandates a maximum average of 48-hour working week, yet compliance across European healthcare systems remains inconsistent. Despite widespread transposition into national legislation, enforcement is inadequate, and junior doctors routinely work 60–70+ hours weekly due to opt-out clauses, weak monitoring, and misaligned institutional incentives. This presentation examines EWTD implementation through an Equity, Diversity, and Inclusion (EDI) lens, drawing on European Junior Doctors (EJD) survey data to demonstrate how working time violations intersect with demographic factors including gender, age, and training stage. In this talk, systemic barriers - inflexible training structures, inadequate rest provisions, and differential exposure to workplace violence and harassment -are analyzed, showing how they disproportionately impact women and marginalized groups, exacerbating work–life imbalance, burnout, and workforce attrition. The discussion integrates EJD’s own gender equality policy with WHO data on physician wellbeing, highlighting elevated rates of burnout and psychological distress among European doctors. This presentation contextualizes these workforce challenges within the specific demands of psychiatry training, where extended hours compound emotional labour and occupational stress. The presentation concludes with evidence-based recommendations for policymakers and training bodies: strengthening enforcement mechanisms for working time regulations, implementing targeted protections for vulnerable trainee cohorts, and embedding EDI principles into workforce planning and postgraduate curricula. These strategies aim to create sustainable training environments that promote the principles of equitable, high-quality psychiatric education across Europe.

**Disclosure of Interest:**

None Declared

